# The 100th Day Case Disc

**Published:** 1992-08

**Authors:** Huw B. Griffith

**Affiliations:** Senior Neurosurgeon, Clinical Director


### The 100th Day Case Disc

**Huw B. Griffith, F.R.C.P., F.R.C.S.**
Senior Neurosurgeon,
Clinical Director

8 years ago, I had what I thought was a good idea. It was to see whether we could carry out disc operations as day cases, something which no-one previously had even contemplated, let alone carried out. We in Frenchay had introduced the operation of microdiscectomy in 1979, and this rapidly replaced the older way of doing disc operations without a microscope. The impulse for day case surgery came from our experiences with operating on doctors. The 4 cm incision and immediate relief from the agonizing sciatica (which was the main indication for surgery in most cases), meant that from a crippled analgesic-swallowing patient before surgery, they were now virtually pain free. The doctors upon whom we operated as patients, of course, immediately responded the next day by saying "when can we go home?" Doctors know perfectly well that nothing extra will then happen and that all that is now required is to stay in bed for a few days. Stretching a wound by moving is painful. The usual run of patients, of course, thought "doctor knows best" and did not make such revolutionary requests! Because this experience was repeated several times, I began to feel that we could contemplate doing the operation on a day case basis.

I now started to ask the patients who had the operation as an inpatient and who were returning to the outpatient clinic 3 weeks or so after surgery, whether, in view of their experience with the operation and the recovery from it, it might be feasible to carry it out as a day case, providing there were somebody to care for them at home and provided their medical and nursing needs were looked after. When we asked those questions of the males (slightly less than 2/3rds of those having disc operations), they said fairly uniformly, "yeah, we think you can!". When we asked the ladies, who form a modest minority, they uniformly said "not on your life!". Do not forget that this operation is usually carried out on patients in their 40's, so that when Mum has surgery she would probably rather be in hospital away from all the demands of husband and children. Hence, I believe, the female reluctance. We have since discovered that where the female patient is going to be looked after well by husband, mother, daughter, son or some other conscientious carer they would rather be at home than in hospital. There was no real difference in the acceptance of the operation. The initial difference was one of social perception.

So I felt we could make a start. My first task was to talk to some of our local GPs who we see at lunch in the Post-Graduate Centre. They thought this might well be a good idea. My main preoccupation was about the need for medical care afterwards and the possibility of post-operative retention of urine which would be a real nuisance if it happened at home. I need not have worried. No patient has had retention of urine even though similar patients having the operation in hospital have frequently had post-operative retention. It is clear that the retention is due to anxiety and tension engendered by the hospital environment, reduced when the patient is snugly tucked up in his own bed at home. The GPs felt that this could be coped with. My compact with them was to say that there would be an implied contract such that were the patient to experience any complication which the GP felt would be best managed in hospital, we would take back the patient immediately without demur or argument.

When we got to the nursing involvement, a different story emerged. I made contact with the lady who ran the District Nursing service and it was apparent from the start that there would have to be a lot of bureaucracy and additional funds before they would agree to do anything. I felt that this was typical organisational Health Service behaviour and was best treated by ensuring that no nursing would be required. This meant that the skin would have to be closed with a suture which did not need removal and be covered by a dressing which the patient could themselves take off after about 10 days. This is what we did and it has worked very well.

What about Physiotherapists? Well, what about them? If patients were going home they did not need "mobilization" in order to be ready to go home. "Mobilization" is the process which occurs in hospital, when we observe a patient in some discomfort being suspended between a brawny physiotherapist on one side and an equally muscular staff nurse on the other. If you are already at home you do not need mobilization.

We started in early 1985 and from the beginning everything went very well. We carried out precisely the same operation as we had done on inpatients. We did not need to modify the procedure in any way. There was, as with inpatients, a very small incidence of "closed disc syndrome", and an eventual reprolapse rate of about 5%. Apart from this, we have had little trouble. One patient was returned to us at about 3 days, but she has been the only one in the first 100. She was a rather excitable lady of continental origin, married to a farmer. His priorities, of course, were riding around on his tractor and not looking after her. She felt neglected, squawked, and her doctor sent her in. We could find nothing wrong, kept her in for 24 hours and sent her home the next day. She made a full recovery.

The regime for the patients was that they stayed in bed for 5 days and mobilized themselves gently by walking, increasing the duration and frequency. By 2½ weeks many of them were ready to go back to work, although they were warned not to put their back under stress, for at this stage the increase of wound strength when healing will not have gone very far. Manual workers were advised not to go back to work for 4 weeks or so.

Beyond telling the patients, if there were post-operative discomfort, to take the same pain-killers prescribed for their sciatica before the operation, we did not offer detailed instructions beyond advice to stay in bed for 5 days. Of course they were to be helped to the toilet near the bathroom for the first 48 hours or so. However, most folk then decided they could go there unaided. When the patient reported back to the clinic, we wished to know how much post-operative pain they had experienced and we were very interested to find that 60 of them took no pain killers at all! Pain, like retention of urine, turns out to be largely a hospital phenomenon as far as this operation is concerned.

Introducing a new procedure like this caused a stir among the nursing staff. I found that one of our excellent ward sisters, (for we took our day patients at first into the ordinary neurosurgical ward) was telling the patients at their departure for home that she was very worried about them! This is understandable, since the post-operative nursing regime in hospital for the first days is to observe the patient carefully, and in particular to test their reactions, strength and sensation in case there were any untoward intraspinal bleeding or indeed any other complication. This made me feel that we should manage these patients in the new day case ward. When the ward was set up, this is what we did. The less threatening environment of a ward where everyone is going to go home seems to be more appropriate. Naturally we have had one or two patients where for good reasons we have felt that it would not be in the patient's best interest to go home. Persistent vomiting after anaesthesia is one of these and has happened three or four times.

Occasionally, despite what we have felt was clear and rigorous selection, a patient creeps in who turns out to have forgotten to tell us about the coronary they had six months previously. By and large however, in this relatively fit age group which coincides with the maximum earning capacity in life, we have had little medical trouble. The operations are carried out in the morning so that we can observe the patients for a minimum of six hours before they return home, just in case bleeding takes place. None has in the first 100 patients. This means that these day case disc patients are the first on the operation list, but this is now clearly understood. The patients usually return home by ambulance. We have set an arbitrary limit of 15 miles radius for distance from Frenchay, but this may well now be extended. We have also set an age limit of 60 years, but this too may go, perhaps under the onslaught of ageism!

We have found it convenient for both doctor and patient to give the patients an information leaflet about what disc prolapse is and what the operation does, added to which guidelines for the recovery period seem to be helpful. In case there is any anxiety in the post-operative phase we give them the telephone number of one of our excellent physiotherapists. She has the occasional telephone call and is able to reassure with effect. We also let the patient's GP know by a separate letter sent out before the operation, together with the patient's information leaflet, what has been arranged and we have had no complaints so far from the family doctors.

We have introduced another little novelty in the last five years or so. When the GP refers the patient we take the history, which contains 80% of the diagnostic information, by telephone since virtually 100% of patients in this age group have telephones. The patient therefore has to make only one visit to hospital. When they come for CT scan, which is our standby for radiological confirmation of the diagnosis, we examine them and are pleased to find that they know us already since we have spent 15 or 20 minutes talking to them on the telephone. If more medical specialties used this technique we should save a great deal of expensive transport and travelling, outpatient parking, outpatient clinic capacity, and perhaps most of all, waiting time for patients and relatives. As we now have the history, the examination, and the CT scan we can make a final decision as to surgery and arrange a date with the outpatient ward. The use of telephone history-taking is a very inexpensive way of doing medicine, and more doctors should practice it. No sophisticated, difficult, or incredibly expensive arrangements or tests are necessary. It has another advantage. When the GP refers the patient, they usually do so by telephone. They report their examination findings, so that when a decision to go ahead and order a CT scan is made, our telephone history with the GPs examination comprises the joint clinical data on which the first decisions are made. This is a real management partnership between hospital consultant and GP. As general practice standards rise steadily more use should be made of this technique. Furthermore, we have discovered that by asking their height and weight over the telephone, we can make a good guess as to whether the patient is too fat for high quality CT pictures, and are enabled to make other arrangements such as MRI scan or even myelography. Again, we have discovered that when we ask the patient to walk on tip-toe, to test plantarflexion power, and then on their heels to test dorsiflexion power we get some surprising and informative results. Some patients discover that they have motor disabilities which lay unsuspected under the blanket of pain and disability which is naturally their main concern. In recent months we have extended this "examination by telephone" to get them to estimate their own straight leg raising angle on either side. They seem to be surprisingly accurate at it.

So two weeks ago we carried out our 100th operation. No-one else except one of our trainees in Delhi, India, seems to have adopted this way of dealing with prolapsed lumbar discs in spite of publishing in medical journals, talking at professional meetings, and generally trying to spread the news, especially by the Sunday newspapers! The fact that this is clearly a highly cost effective way of doing what needs to be done surgically, that the patients like it, and it saves precious hospital beds, has appeared to make absolutely no difference. Why? We have calculated that British neurosurgeons need to do approximately 20 times as many disc operations as are at present being carried out. The need is there. The GPs want this kind of service for their patients. We had hoped that the coming of the Health District/Purchaser would at last result, after due prioritisation, in a contract to carry out the required number (which is easy to calculate - one per 1000 population per annum). We are still waiting.

I still think, and experience has now borne this out, that day case disc surgery is more than a good idea. It is a very good idea. Because I believe that it should be more practised, I have asked Sir Gordon Higginson, who heads the Clinical Standards Advisory Group, to investigate why this extremely inexpensive good idea is not being carried out in large numbers by the Health Service. Perhaps its conclusion will be that this is yet further evidence that the Health Service needs the reforms, and needs to be more responsive to the needs of patients. We shall see.

